# Neural Networks Supporting Phoneme Monitoring Are Modulated by Phonology but Not Lexicality or Iconicity: Evidence From British and Swedish Sign Language

**DOI:** 10.3389/fnhum.2019.00374

**Published:** 2019-10-22

**Authors:** Mary Rudner, Eleni Orfanidou, Lena Kästner, Velia Cardin, Bencie Woll, Cheryl M. Capek, Jerker Rönnberg

**Affiliations:** ^1^Linnaeus Centre HEAD, Swedish Institute for Disability Research, Department of Behavioural Sciences and Learning, Linköping University, Linköping, Sweden; ^2^Deafness, Cognition and Language Research Centre, Department of Experimental Psychology, University College London, London, United Kingdom; ^3^School of Psychology, University of Crete, Rethymno, Greece; ^4^Department of Philosophy, Saarland University, Saarbrücken, Germany; ^5^School of Psychology, University of East Anglia, Norwich, United Kingdom; ^6^Division of Neuroscience & Experimental Psychology, School of Biological Sciences, University of Manchester, Manchester, United Kingdom

**Keywords:** sign language, lexicality, iconicity, semantics, phonology, language processing

## Abstract

Sign languages are natural languages in the visual domain. Because they lack a written form, they provide a sharper tool than spoken languages for investigating lexicality effects which may be confounded by orthographic processing. In a previous study, we showed that the neural networks supporting phoneme monitoring in deaf British Sign Language (BSL) users are modulated by phonology but not lexicality or iconicity. In the present study, we investigated whether this pattern generalizes to deaf Swedish Sign Language (SSL) users. British and SSLs have a largely overlapping phoneme inventory but are mutually unintelligible because lexical overlap is small. This is important because it means that even when signs lexicalized in BSL are unintelligible to users of SSL they are usually still phonologically acceptable. During fMRI scanning, deaf users of the two different sign languages monitored signs that were lexicalized in either one or both of those languages for phonologically contrastive elements. Neural activation patterns relating to different linguistic levels of processing were similar across SLs; in particular, we found no effect of lexicality, supporting the notion that apparent lexicality effects on sublexical processing of speech may be driven by orthographic strategies. As expected, we found an effect of phonology but not iconicity. Further, there was a difference in neural activation between the two groups in a motion-processing region of the left occipital cortex, possibly driven by cultural differences, such as education. Importantly, this difference was not modulated by the linguistic characteristics of the material, underscoring the robustness of the neural activation patterns relating to different linguistic levels of processing.

## Introduction

Natural sign languages can be described using the same linguistic terminology as spoken languages (Sandler and Lillo-Martin, 2006). For example, the term lexicality refers to whether or not an item belongs to the vocabulary of a particular language. This definition applies to both sign languages and spoken languages, indicating theoretical equivalence. Functional equivalence of sign language and spoken language is indicated by the fact that they follow similar developmental milestones (Emmorey, 2002) and show similar neural representation (Rönnberg et al., 2000; MacSweeney et al., 2008). This means that natural sign languages provide a tool for investigating the neural underpinnings of aspects of linguistic processing that are hard to isolate using spoken languages. For example, apparent lexicality effects relating to word processing can be confounded by grapheme-phoneme conversion because lexical access may take place *via* the orthographic route, even when stimulus items are speech recordings (Xiao et al., 2005). Such a strategy is likely to reveal lexicality *via* orthography even in the absence of lexicality effects *via* an auditory route. Sign languages have semantics and phonology but no orthography^1^. Thus, they allow us to investigate the influence of lexicality on language processing without the confounding effects of orthographic processing.

Grosvald et al. (2012) studied the effect of lexicality on sign language processing using a sign language analog of the kind of phoneme-monitoring task that has often been used in studies of spoken language. In the classic phoneme-monitoring task (e.g., Pitt and Samuel, 1995) participants give a button-press response when they recognize a target phoneme in a spoken word or non-word. In the sign-based version introduced by Grosvald et al. (2012), participants responded to short videos of signs and non-signs when the stimulus displayed a target handshape. Handshape is one of three recognized contrastive phonological components of sign language; the others are location and movement (Sandler and Lillo-Martin, 2006). Handshape refers to the form of the signing hand or hands, location refers to the position in space or in contact with the body where the sign is articulated, and movement refers to the path traced by the signing hand or hands. The study by Grosvald et al. (2012) revealed no effect of lexicality on sign processing, as deaf signers showed no difference in the accuracy or latency of their responses to signs and non-signs during handshape monitoring. This finding was in line with Carreiras et al. (2008) who showed no effect of lexicality on handshape priming, although they did report evidence that lexicality influenced location priming. Further, Gutiérrez et al. (2012) reported lexicality effects on ERP modulation elicited by phonological priming; the effects differed for location (modulating the N400) and handshape (modulating a later component), but both handshape and location priming were affected by lexicality. Thus, there is neurophysiological evidence that lexicality influences sublexical processing of sign language relating to handshape and location, and behavioral evidence that it influences sublexical processing of sign language relating to location.

In a recent study (Cardin et al., 2016), we administered a sign-based phoneme-monitoring task (see Grosvald et al., 2012). The stimuli were manual actions that belonged to four different categories: British Sign Language (BSL, lexicalized in BSL but not Swedish Sign Language, SSL); SSL (lexicalized in SSL but not BSL); Cognates (lexicalized in both BSL and SSL, and rated as more iconic than items in the BSL and SSL categories); Non-signs (made-up signs which violated the phonological conventions of both BSL and SSL and were lexicalized in neither). It is important to note that the illegal non-signs used in Cardin et al. (2016) were qualitatively different from the legal non-signs used by Grosvald et al. (2012) as well as by Carreiras et al. (2008) and Gutiérrez et al. (2012). The participants in Cardin et al. (2016) were deaf signers, deaf non-signers and hearing non-signers, all with a British cultural background and so the SSL stimuli were the equivalent of the legal non-signs used in the previous studies (Carreiras et al., 2008; Grosvald et al., 2012; Gutiérrez et al., 2012). The advantage of using signs from a mutually unintelligible sign language instead of made-up signs is that lexicalized and non-lexicalized items are all natural signs.

During fMRI scanning, the participants in the study by Cardin et al. (2016) were cued to monitor the manual actions in each of the four categories for handshape and location in two different versions of the phoneme-monitoring task. The results showed an effect of phonological violation (i.e., a difference between legal signs and illegal non-signs) on the neural networks supporting phoneme monitoring, but no effects related to lexicality (i.e., contrasting lexicalized BSL and non-lexicalized SSL to determine neural activation relating to whether or not stimulus items were part of the participant’s vocabulary) or iconicity (i.e., contrasting Cognates and BSL, which differed in iconicity or the extent to which stimulus items looked like their referents). Indeed, non-signs compared to signs elicited stronger activation in an action observation network in all participants. This indicates greater processing demands for illegal compared to legal signs, irrespective of sign language knowledge and suggests that the phonological characteristics of language may be determined by neural processing efficiency. The absence of a lexicality effect was in line with behavioral work showing no effect of lexicality on handshape monitoring (Grosvald et al., 2012) and imaging work showing no effect of lexicality on processing single signs (Petitto et al., 2000). However, it was not in line with work showing neurophysiological effects of lexicality on handshape processing (Gutiérrez et al., 2012) and both behavioral (Carreiras et al., 2008) and neurophysiological effects of lexicality on location processing (Gutiérrez et al., 2012). Furthermore, the results of Cardin et al. (2016) did not support work showing greater activation in left inferior frontal gyrus for processing signed sentences than pseudosentences consisting of non-linguistic manual actions (MacSweeney et al., 2004) for deaf signers, and greater activation in left angular and supramarginal gyri for processing gestures with meaning compared to those without (Husain et al., 2012). Finally, the results of Cardin et al. (2016) did not support findings relating to speech processing showing differences in neural networks underpinning phoneme monitoring with spoken words and non-words (Newman and Twieg, 2001;Xiao et al., 2005).

In the present study, we honed the experimental design to focus on lexicality. We achieved this by recruiting deaf signers who were native users of either BSL or SSL but who had no knowledge of the other sign language. This allowed us to use BSL as lexical items and SSL as non-lexical items for the BSL signers and vice versa for the SSL signers, thus avoiding the confound of using different materials for these conditions. Cognates and non-signs had the same status for both groups. The tasks and the materials were identical to those used in Cardin et al. (2016).

We predicted that if lexicality does influence the neural networks that support phoneme monitoring, as suggested by previous work showing effects of lexicality phoneme monitoring of spoken words (Newman and Twieg, 2001; Xiao et al., 2005) as well as on sublexical (Carreiras et al., 2008; Gutiérrez et al., 2012) and lexical (MacSweeney et al., 2004) processing of sign language, the fMRI results of our experiment would reveal this. We were also open to possibility that effects of iconicity and phonology on neural networks might differ from those in our previous study because lexicality was involved in both the underlying contrasts. We expected to replicate the significant effect of task observed in our previous study. To confirm our assumption of good experimental control, we tested for main effects of, and interactions with, task and group.

## Materials and Methods

### Participants

Fourteen deaf native British BSL signers and 16 deaf native Swedish SSL signers participated in this study. There were eight women in each group. The data from the British signers are included in group analyses reported in Cardin et al. (2016) that compare signers with non-signers. Data from both British and Swedish signers are included in other analyses comparing signers with non-signers reported in Cardin et al. (2013). The comparison between the two groups of deaf signers from different cultural backgrounds is reported here for the first time. Native signers were defined in the present study as signers with at least one deaf parent who acquired SL from birth through their family. The native language of the British signers was BSL and the native language of the Swedish signers was SSL. All participants stated that they had no knowledge of the non-native sign language used in the study, i.e., SSL for British signers and BSL for Swedish signers, and their familiarity with stimulus items was tested (see “Testing Procedure” section). All participants were screened for deafness by obtaining the pure tone average hearing threshold in decibels (dB) across the frequencies 1 kHz, 2 kHz and 4 kHz (British: *M* = 99.40, *SD* = 8.66; Swedish: *M* = 99.05, *SD* = 11.01) and they were all right handed (Oldfield, 1971).

There was no statistically significant difference in age between groups (British: 29–60, *M* = 38.07, *SD* = 11.91; Swedish: 23–54, *M* = 34.14, *SD* = 10.11, *t* = 0.36, *p* > 0.05). Non-verbal intelligence was assessed using the block design subtest of the Wechsler Abbreviated Scale of Intelligence (British: *M* = 62.36, *SD* = 5.94; Swedish: *M* = 58.57, *SD* = 5.60) and there was no statistically significant difference in block design performance between groups, *t* = 0.10, *p* > 0.05.

### Materials

The materials were identical to those used in Cardin et al. (2016). One-hundred and ninety-two videoclips (2–3 s each) of individual signs were used as experimental stimuli; an additional 48 items were used in a practice session. Items were distributed equally across the four stimulus types: BSL-only (not lexicalized in SSL), SSL-only (not lexicalized in BSL), cognates (signs with the same form and meaning in both languages), and non-signs (sign-like items that have no meaning in either BSL or SSL and which combine phonological parameters in a manner that is either illegal or non-occurring in both languages).

In the present study, SSL stimuli presented to BSL signers and BSL stimuli presented to SSL signers filled the same function as the pronounceable non-signs used in previous studies (Carreiras et al., 2008; Grosvald et al., 2012; Gutiérrez et al., 2012). However, they had the advantages of: (a) being natural rather than constructed signs; and (b) functioning as familiar signs for the other group. Both these factors contributed to good experimental control. Non-signs were included to determine whether the effect of phonological violation reported by Cardin et al. (2016) generalized across sign languages.

All signs were rated for age of acquisition, familiarity, iconicity, and complexity by two native signers of SSL. For BSL and Cognates, age of acquisition, familiarity, iconicity ratings were taken from Vinson et al. (2008) while for SSL these properties, along with the complexity of all signs, were rated by two native signers of BSL. The non-signs were rated for complexity by all four raters. All raters received the same instructions in the appropriate language.

There was no statistically significant difference in complexity ratings across stimulus types or age of acquisition across lexical signs (BSL, SSL, cognates). There was no statistically significant difference in iconicity or familiarity ratings between BSL and SSL signs, but cognates were rated higher on both of these parameters as a natural corollary of their shared visual motivation. Iconicity was described to the raters as occurring when a sign “looks like its meaning,” and both positive and negative examples were given to ensure that the concept was fully understood. The selection of signs is described in full in Cardin et al. (2016).

**Figure 1:**
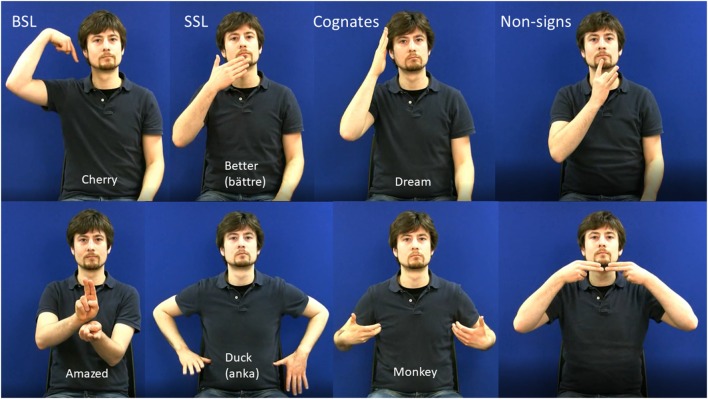
Examples of stills from stimulus videos in all four stimulus categories. The upper panel shows one-handed items and the lower panel shows 2-handed items. English glosses are shown on model’s torso with the Swedish glosses of SSl signs in parentheses.

All experimental stimuli were video-recorded using a high-definition digital camera. The model was a deaf native DGS (German Sign Language) signer who was not a user of either BSL or SSL. This meant that the BSL and SSL signs were equally accented. During recording, the model was visible from the hips to above the head, was seated against a blue background and wore a dark shirt. Examples of experimental stimuli are shown in Figure 1. Apart from the experimental stimuli, there were cues to indicate which version of the task the participant should perform under each condition. The cues indicated one of six phonologically contrastive handshapes or phonologically contrastive locations and consisted of still images.

### Task

A phoneme monitoring task was presented in two versions, one with handshape targets and the other with location targets, cued at the beginning of each block. During blocks of eight stimuli, the participant pressed a button whenever a target stimulus was identified. The behavioral dependent measure was an adapted d’ based on hits adjusted for false alarms in accordance with signal detection theory. Twelve blocks of each of the four stimulus types (BSL, SSL, cognates and non-signs) were presented in randomized order. The participants were asked to fixate on the model’s chin (the lower face is the natural locus of gaze for signers), and a fixation cross was displayed for 500 ms before each stimulus, indicating the location of the model’s chin in the upcoming video. Between stimuli, a blank screen was displayed for an average of 4.5 s, and between blocks, a still of the model with a fixation cross on the chin was displayed for 15 s. This constituted the baseline. Participants were instructed to press the button when the cross changed color from yellow to red.

### Testing Procedure

Before the experiment, the tasks were explained to the participants in their native sign language and written instructions in English or Swedish were provided as appropriate. All participants practiced the tasks before the experiment.

During scanning, participants held the response box in their right hand. Two video cameras in the magnet’s bore were used to monitor the participant’s face and left hand. The experimenter monitored the participant’s face to ensure that he or she was relaxed and awake throughout scanning and the participant’s left hand to determine if the participant wished to communicate with the experimenter. A video camera in the control room allowed the experimenter to communicate with the participant between runs in SSL or BSL using the screen.

After scanning, all signed stimuli used in the experiment were presented to each participant one more time outside the scanner. For each stimulus they indicated whether it was a familiar sign and if so what it meant. Any item which was not correctly categorized was excluded from analysis for the particular individual.

All participants gave their written informed consent and were given ear-protection. This study was approved by the Swedish Regional Ethical committee and the UCL Ethical committee. All participants traveled to Birkbeck-UCL Centre of Neuroimaging in London to take part in the study and were compensated for their travel and accommodation expenses.

### Image Acquisition and Data Analysis

Images were acquired with a 1.5T Siemens Avanto scanner (Siemens, Erlangen, Germany) and a 32-channel head coil at the Birkbeck-UCL Centre for Neuroimaging, London. Functional imaging data were acquired using a gradient-echo EPI sequence (repetition time = 2,975 ms, echo time = 50 ms, field of view = 192 × 192 mm) giving a notional resolution of 3 × 3 × 3 mm. Thirty-five slices were acquired to obtain whole-brain coverage without the cerebellum. Each experimental run consisted of 348 volumes taking approximately 17 min to acquire. The first seven volumes of each run were discarded to allow for T1 equilibration effects. An automatic shimming algorithm was used to reduce magnetic field inhomogeneities. A high resolution structural scan for anatomical localization purposes (magnetization-prepared rapid acquisition with gradient echo, repetition time = 2,730 ms, echo time = 3.57 ms, 1 mm^3^ resolution, 176 slices) was taken either at the end or in the middle of the session.

Out of the total of 192 stimuli presented, data relating to on average 14 (*SD* = 8.5) stimuli were excluded from analysis due to incorrect categorization at post-test. Fewer non-signs were excluded than signs (*p* < 0.01) but there were no overall differences in exclusions between the different categories of signs (all *p*s > 0.05). There were fewer exclusions for Swedish (*M* = 8, *SD* = 2.8; *p* < 0.001) than British signers (*M* = 20, *SD* = 8.6). This difference was largely attributable to the SSL stimuli, all 48 of which were correctly recognized by the Swedish signers but among which the British signers also recognized on average 11 items (*SD* = 5.9) which were thus excluded from analysis for those individuals. The Swedish signers recognized slightly fewer non-signs (*M* = 0.1, *SD* = 0.9, *p* < 0.05) than the British signers (*M* = 1.8, *SD* = 3.0), but there were no significant differences (*p* > 0.05) in exclusions between groups for BSL or Cognates.

Imaging data were analyzed using Matlab 7.10 (Mathworks Inc., MA, USA) and Statistical Parametric Mapping software (SPM8; Wellcome Trust Centre for Neuroimaging, London, UK). Images were realigned, coregistered, normalized, and smoothed (8 mm FWHM Gaussian kernel) following SPM8 standard preprocessing procedures. Analysis was conducted by fitting a general linear model with regressors representing each stimulus type, task, baseline, and cue periods. For every regressor, events were modeled as a boxcar of the adequate duration, convolved with SPM’s canonical hemodynamic response function and entered into a multiple regression analysis to generate parameter estimates for each regressor at every voxel. Movement parameters were derived from the realignment of the images and included in the model as regressors of no interest. Contrasts for each experimental stimulus type and task [e.g., (BSL location > Baseline)] were defined individually for each participant and taken to a second-level analysis.

To test for main effects of group and task, and interaction between task and group, a whole brain analysis of variance (ANOVA) was performed. The factors entered into the analysis were group (British, Swedish), task (handshape, location) and material (BSL, SSL, cognates, non-signs), resulting in a 2 × 4 × 2 ANOVA. Further analyses (described under the relevant parts of the “Results” section) were performed to isolate effects of Iconicity, Semantics, and Phonology. Significant activations at *p* < 0.05 corrected (FEW) at cluster or peak level are reported. Voxels are reported as x, y, z coordinates in accordance with standard brains from the Montreal Neurological Institute (MNI).

## Results

### Behavioral Data

**Figure 2:**
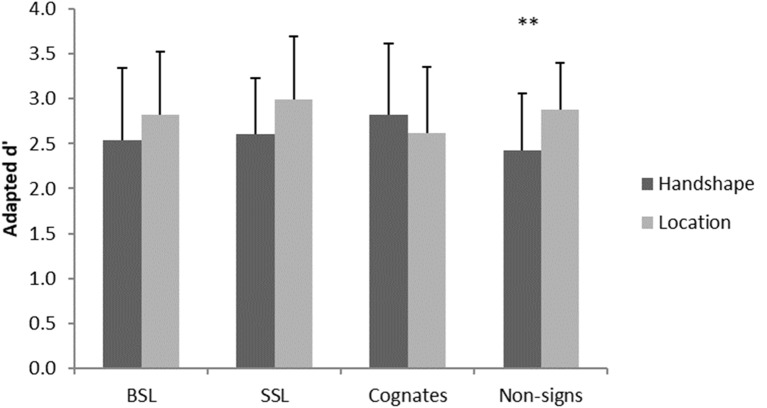
Significant interaction in behavioral performance across groups. Error bars show standard deviation. **p < 0.01.

Data were analyzed by calculating a mixed repeated-measures ANOVA based on a 2 × 2 × 4 design with the factors group (British, Swedish), task (handshape, location) and material (BSL, SSL, cognates, non-signs). There was no significant main effect of group, *F*_(1,26)_ = 0.00, *p* = 0.99 or material, *F*_(3,78)_ = 0.84, *p* = 0.48. However, there was a significant main effect of task, *F*_(1,26)_ = 4.90, *p* = 0.036, and a significant interaction between task and material, *F*_(3,78)_ = 5.11, *p* = 0.003. Investigation of this interaction using paired sample *t*-tests with Bonferroni correction for multiple comparisons showed better performance on the location than handshape task for non-signs, *t*_(27)_ = 3.92, *p* = 0.004, while there was no significant difference in performance between tasks with any of the other materials (all *p*s > 0.05), see Figure 2.

### fMRI Data

#### Effect of Group

**Figure 3:**
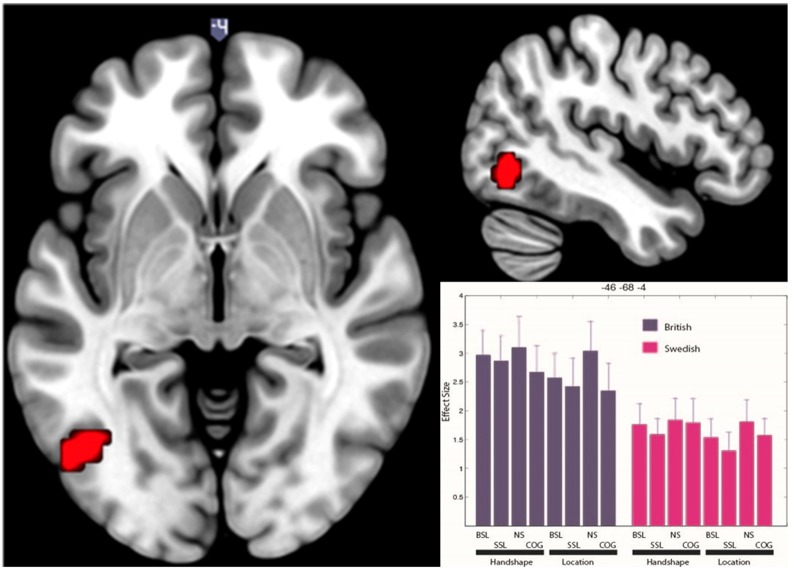
Significantly greater BOLD response in visual motion-processing regions of the left occipital cortex for British deaf native signers than Swedish deaf native signers. The figure shows clusters significantly active (*p* < 0.05 FWE) for the contrast (BSL signers > SSL signers) across all conditions. The activation is rendered on the standard MNI brain. The axial slice is at *z* = −4, while the sagittal slice is at *x* = −46. The histogram shows effect size in each condition (NS, non-signs; COG, cognates) for each group at −46 −68 −4. Error bars show standard error of mean.

In the main ANOVA, there was no significant net activation for deaf native signers of SSL compared to deaf native signers of BSL. However, in the British group, compared to the Swedish group, there was significantly more activation across all conditions in a motion-processing area of the left occipital cortex (−46 −68 −4; see Figure 3). This cluster appears near the visual motion area MT/V5 (Tootell et al., 1995). We used SPM Anatomy Toolbox (Eickhoff et al., 2005) to determine the location of this cluster in relation to a probabilistic map of area MT/V5 (Malikovic et al., 2007). The results of this analysis revealed that the activation cluster is anterior and dorsal to MT/V5 (Eickhoff et al., 2005), with 10–30% chance of being within this cytoarchitectonic area. This suggests that this activation is located within, or anteriorly to, the MT+ complex, which encompasses V5/MT and other motion-sensitive areas, and likely corresponds to a higher-order visual motion region (Kolster et al., 2010).

#### Effect of Task

The handshape task minus the location task generated more activation in bilateral regions including the intraparietal sulcus (30 −55 46, −48 −37 46), the ventral occipito-temporal cortex (−24 −70 −8, 36 −82 4) and the inferior frontal gyrus (−45 5 31, 48 8 31). The location task compared to the handshape task generated more activation in the cingulate sulcus visual area (−39 −76 31, 48 −76 25) and right angular gyrus (12 −55 19). This pattern of results from a total of 28 deaf signers (Swedish and British) is similar to the pattern reported previously for 15 British deaf signers (Cardin et al., 2016).

#### Effects of Lexicality, Iconicity and Phonology

##### Lexicality

The effect of lexicality was examined by investigating neural activation for familiar signs minus unfamiliar signs across groups and tasks. For the British group, familiar signs were BSL and unfamiliar signs were SSL, while for the Swedish group, familiar signs were SSL and unfamiliar signs were BSL. There was no significant main effect of lexicality (or the opposite contrast) and there were no significant interactions with either task or group.

##### Iconicity

The effect of iconicity was examined by investigating neural activation for cognates (same for both groups) minus familiar signs (BSL for the British group and SSL for the Swedish group) across tasks. The cognate stimuli had a shared visual motivation across languages and were thus rated as being more iconic than the language-specific stimuli. There was no significant main effect of iconicity (or the opposite contrast) on neural activation and there were no significant interactions with either task or group.

##### Phonology

The effect of phonology was examined by investigating neural activation for unfamiliar signs (SSL for the British group and BSL for the Swedish group) minus non-signs (same for both groups). Because the phonological inventories of BSL and SSL are similar, even the unfamiliar signs are likely to match existing phonological representations. There was no significant main effect of phonology. However, the opposite contrast showed activation in the supramarginal gyrus bilaterally (34 −46 49, −57 −25 41, 55 32 30, −31 −87 23) as well as in the left parietal lobule (−35 −42 46, −17 −63 53) and superior frontal gyrus (−24 −2 61), in agreement with Cardin et al. (2016). No significant interaction with task or group was found with either contrast.

## Discussion

In the present study, we investigated the effects of lexicality, iconicity and phonology on phoneme monitoring. We used sign language to avoid the confounding effect of orthographic recoding associated with spoken language and crossed materials across groups to avoid the confounding effect of using different materials for lexical and non-lexical items. fMRI results showed no significant effect of lexicality or iconicity on the neural networks associated with phoneme monitoring, and the effect of phonological violation agreed with previous work (Cardin et al., 2016). In addition, we found that a motion-processing region of the left occipital cortex was activated more by the British signers than the Swedish signers during phoneme monitoring, irrespective of material or task.

### Lexicality

In the present study, lexicality was operationalized as the contrast between BSL and SSL, where BSL signs constituted lexical items and SSL non-lexical items for the BSL signers and vice versa for the SSL signers. This meant that both BSL and SSL signs occurred as both lexical items and non-lexical items making for good experimental control. The results of the present study revealed no effect of lexicality on the neural networks supporting phoneme monitoring (handshape and location). This is in line with Grosvald et al. (2012) who showed no effect of lexicality on speed or accuracy of handshape monitoring and Petitto et al. (2000) who showed no effect of lexicality on the neural networks supporting passive observation of single signs. It also extends our own previous work (Cardin et al., 2016) by showing that the lexicality effect is absent during phoneme monitoring even when possible effects of materials are controlled for and power is increased. Further, it shows that the effect generalizes from BSL to another sign language—SSL. Moreover, fewer items had to be excluded from analysis for Swedish than British signers, increasing the reliability of the present analysis.

However, the findings of the present study are at odds not only with studies showing an effect of lexicality on the neural networks underpinning phoneme monitoring of spoken words and non-words (Newman and Twieg, 2001; Xiao et al., 2005) but also studies that have shown an effect of lexicality on sublexical processing of sign language (Carreiras et al., 2008; Gutiérrez et al., 2012) as well as studies that have reported an effect of meaningfulness on the neural networks supporting processing of manual actions (MacSweeney et al., 2004; Husain et al., 2012).

The studies by Carreiras et al. (2008, Experiment 3) and Gutiérrez et al. (2012) required the participants to make a lexical decision on a target sign or pronounceable non-sign preceded by a phonologically related or unrelated item. Thus, the task had a direct bearing on the lexicality of the target item. The task used in the study by MacSweeney et al. (2004), although not directly related to lexicality, required participants to monitor for semantic anomaly, i.e., non-meaningfulness, among BSL sentences and strings of manual actions belonging to a non-linguistic manual-brachial code known as TicTac sometimes used by bookmakers at racecourses. In the study by Husain et al. (2012), participants performed a delayed match to sample task in which they were instructed in one version to determine whether the target gesture was identical to the sample gesture, and in the other version to determine whether the target belonged to the same category as the sample, the two possible categories being emblematic gestures and meaningless gestures. Thus, the task used by Husain et al. (2012) also tapped into meaning.

To sum up, the studies showing an effect of lexicality or meaningfulness on sign language processing (MacSweeney et al., 2004; Carreiras et al., 2008; Gutiérrez et al., 2012; Husain et al., 2012) all included at least one task that specifically draws attention to lexicality or meaningfulness. This differs from the present study which used a task based on monitoring phonological features of the stimuli, in line with Cardin et al. (2016) and Grosvald et al. (2012), with no requirement to take into account either lexicality or meaningfulness. No sign-based phoneme monitoring study to our knowledge has found effects of lexicality on sign language processing. Taken together, the evidence suggests that lexicality does not influence sign language processing at the sub-lexical level when meaningfulness is not in focus, and supports the notion that apparent lexicality effects relating to spoken word processing actually reflect the grapheme-phoneme conversion required when lexical access takes place *via* the orthographic route (Xiao et al., 2005).

### Iconicity

In the present study, iconicity was operationalized as the contrast between, on the one hand, Cognates and, on the other hand, BSL signs for the BSL signers and SSL signs for the SSL signers. Iconicity was operationalized in this way because given the lack of common ancestry between BSL and SSL, the Cognates were by definition iconic signs by virtue of meaning having been incidentally mapped to the same surface representation in both languages. The greater iconicity of the Cognates compared to the stimuli consisting of signs occurring only in BSL or only in SSL was demonstrated by the significant difference in iconicity ratings. No effect of iconicity was revealed by the present study. This finding is in line with our previous study (Cardin et al., 2016) and generalizes it from BSL to SSL using the very same materials. It is also in line with the well-established notion that the link between a lexical item and its referent is characterized by its arbitrariness, and that any surface resemblance is irrelevant and does not influence language processing (for a review, see Perniss et al., 2010). However, it deviates from a recent set of findings suggesting that iconicity does indeed influence language processing and that under certain circumstances it may provide a link to experience that helps bridge the gap between linguistic form and conceptual representation (Perniss et al., 2010). In particular, Thompson et al. (2010) showed that iconicity interferes with phonological decision-making, rendering it slower and less accurate. However, Emmorey (2014) argued that psycholinguistic effects of iconicity may only be observed when the task specifically taps into the structured mapping capturing the resemblance between the form and its meaning. The task employed by Thompson et al. (2010) involved determining whether the fingers in any particular sign were straight or curved, tapping into structural mappings to objects with flat sides (e.g., BSL BOX) or curved sides (e.g., BSL CUP) respectively. The tasks used in the present study, however, which involved determining whether the handshape or location of each sign presented matched that of a given target, did not specifically tap into structural mappings.

### Phonology

In the present study, phonology was operationalized as the contrast between legal signs and illegal non-signs. Phonologically illegal items elicited stronger activation in an action observation network than phonologically legal signs, in agreement with Cardin et al. (2016). This network included the supramarginal gyrus, a region associated with phonological processing in both sign language and spoken languages. This finding strengthens the notion of greater processing demands for illegal compared to legal signs (whether actual signs or phonologically possible signs), by showing that it generalizes across sign languages and is independent of the specific materials used as legal signs. Interestingly, behavioral results showed better performance on the location than handshape task for non-signs, i.e., when manual actions were phonologically illegal, but not for any of the legal signs. This suggests that the perceptual salience of location over handform (Brentari et al., 2011) plays out to a greater extent for illegal manual actions than legal signs, strengthening the notion that phoneme monitoring of illegal items engenders perceptual rather than phonological processing.

### Task

The phoneme monitoring task used in the present study was administered in two versions, one with handshape cues and one with location cues. Differences in the perceptual processing of handshape and location also became apparent in neural activation. Handshape compared to location monitoring generated more activation in the ventral visual stream while the opposite contrast generated more activation in the dorsal visual stream. This is in line with the perceptual nature of the two versions of the task: the handshape version focusing on “what” and the location version on “where” (Milner and Goodale, 1993; Ungerleider and Haxby, 1994). It also chimes in with the finding that location priming modulates an earlier ERP component (N400) than handshape (Gutiérrez et al., 2012).

### Group

The main effect of group on the neural correlates of sign-based phoneme monitoring was novel and unexpected. In particular, the left occipital cortex, close to area V5/MT+ was activated more by the British signers than the Swedish signers during phoneme monitoring. This region is likely to be part of the MT+ complex, and could be involved in the kind of complex motion processing necessary to determine the phonological characteristics of manual gestures. However, because the phonological inventories of BSL and SSL are highly similar, it is unlikely that there are systematic differences in the motion processing demands of the experimental tasks across languages. Fewer items had to be excluded from the analysis for Swedish than British signers, principally because the British signers unexpectedly recognized some of the SSL signs. We have shown that lexicality does not influence the neural networks supporting phoneme monitoring and thus it is unlikely that the difference in exclusion rate contributes to the observed group effect. The one previous imaging study (Petitto et al., 2000) including deaf users of two sign languages did not report differences in processing between the two groups. Unlike the sign languages used in the present study, BSL and SSL, the sign languages used in the study by Petitto et al. (2000), American Sign Language (ASL) and Quebec Sign Language (LSQ), are historically related, with the former heavily influencing the latter. Thus, the present study is the first to report differences in the neural networks supporting sign processing between well-matched groups of users of distinct sign languages with no known historical links.

Similar regions have previously been found to be activated during sign language processing, including signed sentences and discourse (Söderfeldt et al., 1994, 1997; MacSweeney et al., 2002, 2004, 2006; Emmorey et al., 2014) and individual signs (Petitto et al., 2000; MacSweeney et al., 2006; Capek et al., 2008). The V5/MT+ complex is not generally associated with speech processing (Scott and Johnsrude, 2003; Hickok and Poeppel, 2007) and it has been shown to be activated more by sign than audiovisual speech in hearing signers (Söderfeldt et al., 1997; Emmorey et al., 2014) and more by sign than visual speechreading in deaf signers who are also proficient speech readers (Capek et al., 2008). Thus, the V/MT+ complex seems to play a sign-specific role in language processing that may well involve mapping of visuo-dynamic input to higher-order phonological and semantic representations.

Further, Capek et al. (2008) showed that an adjacent area (−47, −59, −10) was activated more for signs with non-speech-like than speech-like mouth actions in deaf native signers, thus further attesting to the specificity of this region for sign over speech. In that study, speech-like mouth actions (mouthings) were represented by mouth actions that disambiguate minimal pairs of lexical items with identical manual forms and which resemble the equivalent spoken forms (Sutton-Spence and Woll, 1999). Non-speech-like mouth actions (mouth gestures) were represented by one type of mouth gesture, echo phonology, i.e., mouth actions that “echo” on the mouth certain articulatory movements of the hands (Woll, 2014).

Group-specific differences in motion-processing regions of the occipital cortex are also documented in the literature. These include more activation during observation of sign language for hearing signers than hearing non-signers (MacSweeney et al., 2006), indicating sensitivity to the linguistic content of the stimuli; and more activation for hearing than deaf native signers during comprehension of signed sentences, possibly indicating sensitivity to differences in sign language skill (MacSweeney et al., 2004). To summarize, the literature suggests that motion-processing regions of the occipital cortex support the processing of communicative gestures and that their engagement is modulated by the sign-specificity of accompanying mouth actions as well as the ability to access linguistic content and skill in achieving this.

Data from a previous study from our lab comparing memory processing in deaf signers and hearing non-signers in British and Swedish participants indicated that whereas a sign-based memory strategy was used by Swedish participants, a speech-based strategy was used by British participants (Andin et al., 2013). In that article, we argued that this might be because sign language education has been more consistently implemented in deaf education in Sweden (Svartholm, 2010; Andin et al., 2018) than in Britain and that systematic differences in the education models might lead to different sign-processing strategies. Thus, one explanation of the difference in neural networks supporting sign-based phoneme monitoring in British and Swedish deaf signers could be systematic differences in the sign-processing strategies used for task solution (see MacSweeney et al., 2004) due to differences in approaches to the use of spoken language in deaf educational settings. Such differences would be unlikely to occur between sign languages in similar cultural settings (see Petitto et al., 2000).

Bearing in mind that Capek et al. (2008) found differences in the activation of motion-processing regions of the occipital cortex depending on whether the signs observed by the deaf participants included speech-like or non-speech-like mouth actions, it needs to be considered whether the activation difference in the present study is driven by systematic differences in the use of such characteristics between BSL and SSL. Even though the signed stimuli used in the present study did not include mouth actions, it is not inconceivable that representations of mouth actions could be activated during observation and processing of manual actions. Crasborn et al. (2008) compared the distribution of mouth actions in BSL and SSL and found that they were highly similar across languages. In particular, occurrence of mouthings in the corpus examined was 51% in BSL and 57% in SSL while the corresponding occurrence of echo phonology was 2% and 7%. This suggests that even if the two groups of participants had different strategies regarding the extent to which they made use of existing representations of mouth actions while performing the phoneme monitoring task, it is unlikely that such strategy differences would be confounded by systematic differences in the occurrence of different types of mouth actions across these two languages. Thus, we suggest that greater activation of motion-processing regions of the occipital cortex for British compared to Swedish deaf signers during a sign-based phoneme-monitoring task in the present study may be due to the use of different strategies. It is possible that different strategies are driven by different educational experiences, but this needs to be investigated further.

## Conclusion

The pattern of results in the present study suggests that lexicality does not influence sublexical processing of sign language in the absence of lexical task demands. This finding supports the notion that the effects of lexicality previously observed in phoneme monitoring of speech may reflect an orthographic strategy. Further, results showed that the neural networks supporting linguistic processing are modulated by phonological constraints but not iconicity, at least in the absence of structural mapping requirements. Although deaf signers from different language and cultural backgrounds engage largely similar neural networks during sign-based phoneme monitoring, we identified differential activation of motion-processing regions of the occipital cortex possibly relating to differences in strategies possibly driven by cultural differences such as schooling. Importantly, the group effect does not interact with lexicality, underscoring the robustness of the absence of a lexicality effect.

## Ethics Statement

This study was carried out in accordance with the recommendations of the UCL Ethical committee and Swedish legislation with written informed consent from all subjects. All subjects gave written informed consent in accordance with the Declaration of Helsinki. The protocol was approved by the UCL Ethical committee and the Regional Ethical Review Board in Linköping, Sweden.

## Author Contributions

The study was designed by MR, EO, BW, CC and JR. The data were collected by EO, LK, VC and CC and analyzed by LK, VC. MR prepared the first draft of the article and all authors contributed to the final version.

## Conflict of Interest

The authors declare that the research was conducted in the absence of any commercial or financial relationships that could be construed as a potential conflict of interest.

## Footnotes

^1^Some writing systems do exist (e.g., SignWriting), but these are not in general use in any sign language.
